# Older Adolescents and Young Adults With Autism Spectrum Disorder Have Difficulty Chaining Motor Acts When Performing Prehension Movements Compared to Typically Developing Peers

**DOI:** 10.3389/fnhum.2018.00430

**Published:** 2018-10-23

**Authors:** Takao Fukui, Misako Sano, Ari Tanaka, Mayuko Suzuki, Sooyung Kim, Hiromi Agarie, Reiko Fukatsu, Kengo Nishimaki, Yasoichi Nakajima, Makoto Wada

**Affiliations:** ^1^Department of Rehabilitation for Brain Functions, Research Institute, National Rehabilitation Center for Persons with Disabilities, Tokorozawa, Japan; ^2^Information and Support Center for Persons with Developmental Disabilities, National Rehabilitation Center for Persons with Disabilities, Tokorozawa, Japan; ^3^Department of Medical Treatment III (Pediatric and Child Psychiatric Section), Hospital, National Rehabilitation Center for Persons with Disabilities, Tokorozawa, Japan

**Keywords:** reach-to-grasp movements, kinematics, grip aperture adjustment, chaining motor acts, autism spectrum disorders (ASD)

## Abstract

It is known that motor actions performed by individuals with autism spectrum disorders (ASD) are clumsy and a previous study revealed that children with ASD of around 8 years old showed less smooth movement and dysfunction of appropriate usage of online vision for grip aperture control. The present study investigates whether and how the kinematic properties of reach-to-grasp movements in older adolescents and adults with ASD [mean (±SD) age: 18.3 ± 2.1] differ from those in typically developing (TD) peers [mean (±SD) age: 19.1 ± 2.2]. Revealing the kinematic properties of reach-to-grasp movements in older adolescents and adults with ASD is indispensable in determining the developmental trajectory of this motor behavior in individuals with ASD. While wearing liquid crystal shutter goggles, participants reached for and grasped a cylinder with a diameter of either 4 or 6 cm. Two visual conditions were tested: a full vision (FV) condition (the goggles remained transparent during the movement) and a no vision (NV) condition (the goggles were closed immediately after the movement was initiated). These two visual conditions were either alternated with each trial in a single experimental session (alternated condition) or blocked within the session (blocked condition). We found that the reaching movement smoothness calculated as a normalized jerk score (i.e., index of skilled, coordinated human movements) of ASD participants did not differ significantly from that of TD peers although ASD participants showed smoother reaching in the alternated condition than in the blocked condition. The influence of online vision and its visual condition schedule on grip aperture during the in-flight phase was remarkably similar between the ASD and TD groups. Furthermore, we found that ASD group experienced a significant longer transition period from grasping end (i.e., stable holding when touching the surface of the object) to uplift initiation than the TD group. The results suggest that (1) deficits in movement smoothness and the use of online vision for motor control are rectified by the time individuals with ASD reach late adolescence and (2) older adolescents and adults with ASD still have difficulties chaining motor acts.

## Introduction

Autism spectrum disorder (ASD) was first identified by Kanner (1943) and Asperger (1944). Although its etiology is not yet fully known, this developmental disorder is characterized by impairments in social interaction, communication, and imagination (Wing and Gould, 1979; Wing, 1981; American Psychiatric Association, 2000). ASD has traditionally been regarded as a social and cognitive disorder (e.g., Frith et al., 1991; Baron-Cohen and Belmonte, 2005; Happé and Frith, 2006; Senju and Johnson, 2009, for reviews). However, researchers have also explored how people with ASD are different from typically developing (TD) peers in terms of sensory processing (see Iarocci and McDonald, 2006; Ben Shalom, 2009; Bhat et al., 2011; Marco et al., 2011, for reviews) and motor behaviors (see Leary and Hill, 1996; Ben Shalom, 2009; Elliott et al., 2010; Fournier et al., 2010; Bhat et al., 2011; von Hofsten and Rosander, 2012; Fabbri-Destro et al., 2013; Gowen and Hamilton, 2013; Whyatt and Craig, 2013; Sacrey et al., 2014; Cook, 2016, for reviews).

The prospect for early detection of motor abnormalities in infants with ASD is still controversial (Teitelbaum et al., 1998; Ozonoff et al., 2008), but movement disturbances in children with ASD have been found in various motor behaviors, including postural balance (e.g., Kohen-Raz et al., 1992; Molloy et al., 2003), gait (e.g., Vilensky et al., 1981), and hand/arm movements (e.g., Schmitz et al., 2003; Haswell et al., 2009). Some researchers have argued that individuals with ASD (including school-age children and adolescents) have a normal ability to execute movements but showed atypical properties in movement preparation and planning (Hughes, 1996; Rinehart et al., 2001). However, this is still controversial.

Among the various hand/arm motor behaviors, the reach-to-grasp movement is fundamental to daily life and, since Jeannerod’s (1981, 1984) pioneering studies, has been extensively explored in adults (see Fukui et al., 2006; Castiello and Begliomini, 2008; Filimon, 2010; Grafton, 2010; Rosenbaum et al., 2012; Gaveau et al., 2014; Turella and Lingnau, 2014, for reviews in the past decade). Other studies have investigated infants (e.g., von Hofsten and Ronnqvist, 1988; Newell et al., 1989; Corbetta and Thelen, 1995) and children (e.g., Kuhtz-Buschbeck et al., 1998; Smyth et al., 2004; Zoia et al., 2006).

Mari et al. (2003) were the first to investigate the kinematic properties of the reach-to-grasp movements in children with ASD beyond simply the standardized test batteries (e.g., the movement assessment battery for children, Henderson and Sugden, 1992). The authors found that children with ASD (aged 7–13 years) showed longer movement durations, longer deceleration times, lower peak wrist velocities, and longer times to peak grip aperture (PGA) than age-matched control participants, though they noted no significant difference in PGA between the ASD and age-matched control groups. They further suggested that performance in the ASD group could be differentiated according to IQ, finding that children with lower IQ scores (IQ: 70–79) exhibited abnormal coordination between reach and grasp components in slower motor behavior, while children with average and higher IQ score group (IQ: 80–109) showed normal, or even “superior” motor behavior, compared to age-matched control group. Recently, Campione et al. (2016) investigated younger children (aged 4.3–5.9 years) with ASD and no intellectual disability (full-scale IQ >80), and obtained results that were generally consistent with those of Mari et al. (2003).

Yang et al. (2014) also investigated the kinematic properties of reach-to-grasp movements in children with ASD [mean (SD) age: 7 years 8 months (1 year 4 months)] by manipulating online vision during the movement [i.e., full vision (FV) and no vision (NV) conditions]. The classical finding that PGA in the NV condition was significantly larger than that in the FV condition in adults (e.g., Wing et al., 1986; Jakobson and Goodale, 1991; Fukui and Inui, 2006) is well known and has been partially confirmed in children (Kuhtz-Buschbeck et al., 1998; Smyth et al., 2004; Zoia et al., 2006). Yang et al. (2014) found that the contribution of online vision to grip aperture adjustment was smaller in participants with ASD, indicating a significantly larger PGA (compared to that in the control groups) even when online vision was available during movement (i.e., the FV condition). The authors also recorded normalized jerk scores (NJSs), which indicate an extent of movement smoothness (i.e., index of skilled, coordinated human movements) and found that the movement in ASD group was longer (slower) and less smooth than that in the control group, especially when children were reaching for and grasping a smaller target without online vision (i.e., the NV condition).

Recently, it has been demonstrated that grip aperture control can be modulated by the presentation order of trials of FV and NV conditions in healthy adults (Whitwell et al., 2008; Tang et al., 2014, 2015). Specifically, the difference in PGA between FV and NV conditions is smaller when these trials are intermixed in an experimental session than when they are blocked separately. The authors named this effect “homogenization” and argued that the homogenizing effects are “mediated by movement-specific memories that operate over iterations of the same action” (Tang et al., 2015, p. 62). One of the aims of the current study is to test whether homogenization is typical or atypical in adolescents and adults with ASD.

Reach-to-grasp movements are usually performed with a subsequent motor act, depending on a final goal of the action (e.g., Marteniuk et al., 1987; Johnson-Frey et al., 2004). Therefore, proper chaining of motor acts as an entire action is essential for appropriate performance. Fabbri-Destro et al. (2009) investigated how ASD children [including early adolescents (mean age: 10.0 ± 2.3)] and TD peers (matched by non-verbal cognitive level) performed tasks requiring them to reach for and grasp a metal object on a plate, and then pick up and place (drop) it into a container on the right side of the plate. Task difficulty was manipulated by the size of the container (i.e., big vs. small). The authors found that, unlike in the TD group, the reach-to-grasp movements in the ASD group were not appropriately modulated by the subsequent motor act [i.e., placing (dropping)]. Therefore, the authors concluded that the children with ASD had difficulties in chaining motor acts as an entire action (see also Cattaneo et al., 2007; Forti et al., 2011).

The above studies regarding the kinematic properties of reach-to-grasp movements in individuals with ASD were focused on pre- and early adolescent children. No study has yet explored how older adolescents and adults with ASD perform simple reach-to-grasp movements, although pointing movements (without grasping) (Glazebrook et al., 2006, 2009) or more complex motor actions, including in social contexts such as passing a tool to another person after holding it (Gonzalez et al., 2013) have been studied in young adults with ASD. When performing pointing movements, Glazebrook et al. (2009) demonstrated that compared to TD peers, young adults with ASD showed (i) longer reaction time regardless of online vision availability and (ii) longer execution time, especially when vision is available during movement. Furthermore, Gonzalez et al. (2013) showed how people with ASD pass a tool to another person, which is quite different from how TD peers pass a tool to another person. In addition to previous kinematic findings regarding simple pointing movements and social motor action, elucidating the kinematic properties of planning and execution processes in reach-to-grasp movements in older adolescents and adults with ASD is indispensable for uncovering the developmental trajectory of this motor behavior in individuals with ASD. Such elucidation could support the development of prospective therapeutic interventions for movement disturbance.

In this study, we used motion capture system to investigate whether and how the kinematic properties of reach-to-grasp movements in older adolescents and adults with ASD differ from those of TD peers. Based on the studies mentioned above, we tested a task requiring individuals to reach to grasp the object and lift it up after holding it based on three functions: (1) movement smoothness, (2) grip aperture control modulated by availability of online vision (i.e., FV or NV during the movement) and its presentation order, and (3) chaining of motor acts (as an entire action). Specifically, in comparison with TD peers, we focused on (1) whether older adolescents and adults with ASD show clumsy movement (i.e., larger NJS), (2) whether people with ASD could use online vision and show a “homogenization” effect in relation to grip aperture control, and (3) whether people with ASD show a deficit in terms of chaining motor acts as an entire action (i.e., longer time difference between the grasp-end time and the time of lifting initiation).

## Materials and Methods

### Participants

The experiment involved 12 individuals with ASD [one female, mean (±SD) age: 18.3 ± 2.1 years] and 12 TD individuals [one female, mean (±SD) age: 19.1 ± 2.2 years] (Table 1). All participants were right-handed, as assessed by the Edinburgh Handedness Inventory (Oldfield, 1971) and had normal or corrected-to-normal vision. They were naive regarding the purpose of the experiment and were paid for their participation. Since the task involved minimal verbal demands of the participants, the participant groups were matched with respect to non-verbal IQ (Mosconi et al., 2015; Wang et al., 2015), in addition to age, sex, and handedness. IQ assessments were carried out using a Japanese version (Fujita et al., 2006) of the Wechsler Adult Intelligence Scale-III (WAIS-III; Wechsler, 1997) and all participants’ non-verbal IQ scores were higher than 80. The mean verbal IQ in the ASD group was 102.2 (76–134, Table 1), so the participants with ASD and the TD participants were able to understand the task instructions correctly and were confirmed to be capable of following those instructions during the experiment.

**Table 1:** Demographic characteristics of participants with ASD and typically developing peers.

		Age	IQ			AQ	ADOS-2 (Module 4)		
					
			Full	Non-verbal	Verbal		Comm.	SI	Comm. + SI
ASD	MEAN	18.3	100.1	97.7	102.2	27.3	3.4	6.2	9.6
	*SD*	(2.1)	(13.0)	(10.4)	(16.2)	(8.1)	(1.4)	(2.1)	(2.8)
TD	MEAN	19.1	110.6	102.8	114.8	19.1			
	*SD*	(2.2)	(12.1)	(10.1)	(15.2)	(4.6)			
		*t*(22) = -0.847	*t*(22) = -2.044	*t*(22) = -1.213	*t*(22) = -1.976	*t*(22) = 3.060			
		*p* = 0.406	*p* = 0.053	*p* = 0.238	*p* = 0.061	^∗^*p* = 0.006			


*^∗^*p* < 0.05 (independent-samples *t*-test for comparison of ASD and TD groups). Comm., communication score (cutoffs: 3/2); SI, social interaction score (cutoffs: 6/4); Comm. + SI, summed score (communication and social interaction) (cutoffs: 10/7). The cutoffs shown above in parentheses denote the minimum scores for diagnosing autism and autism spectrum disorder, respectively.*

A Japanese version (Wakabayashi et al., 2004) of the autism–spectrum quotient (AQ) test (Baron-Cohen et al., 2001) confirmed that none of the participants in the TD group had clinically significant levels of autistic traits, since each participant’s AQ score was less than the cutoff score (i.e., 33 in the Japanese version). All participants with ASD were diagnosed according to Diagnostic and Statistical Manual of Mental Disorders, 4th edition (DSM-IV; American Psychiatric Association, 2000) or 5th edition (DSM-V; American Psychiatric Association, 2013) by child psychiatrists. Their diagnoses were also assessed by a Japanese version (Kuroda and Inada, 2015) of Autism Diagnostic Observation Schedule Second Edition Module 4 (ADOS-2 Module 4; Lord et al., 2012). Although one participant in the ASD group was classified as non-spectrum by ADOS-2 (Module 4) criteria, this participant was diagnosed by a child psychiatrist; therefore, the participant was included in the ASD group. The exclusion of this participant did not alter the pattern of significance.

The study was approved by the institutional ethics committee at the National Rehabilitation Center for Persons with Disabilities, and all the participants (and their parents, for participants younger than 20 years old) provided written informed consent according to institutional guidelines conforming to the Declaration of Helsinki.

### Apparatus

**FIGURE 1:**
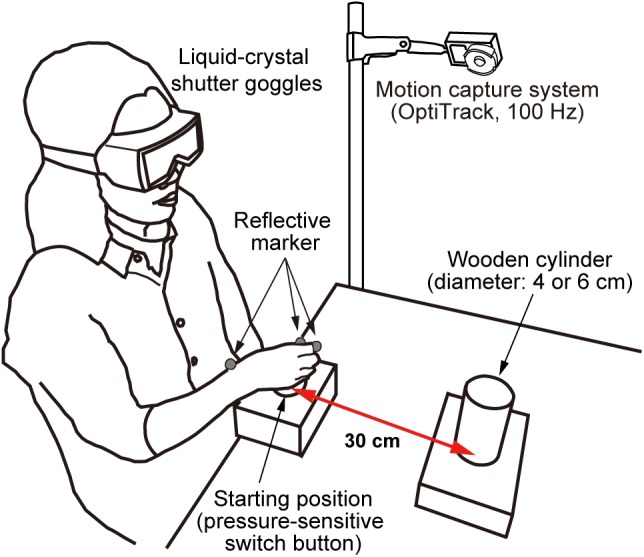
Configuration of the experimental apparatus. Participants wore liquid-crystal shutter goggles and rested their hand on a pressure-sensitive switch button (a starting position). Two wooden cylinders measuring 4 and 6 cm in diameter and 11 cm in height were used as targets in the task. The motion of the reflective markers was recorded by the motion capture system.

As shown in Figure 1, participants wore liquid-crystal shutter goggles (Takei Scientific Instruments Co., Ltd.) while seated comfortably on a chair in front of a table. The shutter goggles, which were also used in our previous studies (e.g., Fukui and Inui, 2006, 2015), take about 3 ms to become transparent and about 20 ms to become opaque. In the starting position, a pressure-sensitive switch button (diameter: 5 cm) was located in line with the participant’s mid-sagittal plane. The center of target object was positioned 30 cm from the center of the switch button. Two wooden cylinders, measuring 4 or 6 cm in diameter and 11 cm in height [weight: 51 g (4 cm), 136 g (6 cm)], were used as targets for the task. Hand movement (monitored by reflective marker attached to the tips of the thumb and index fingers and the dorsodistal aspect of the radial styloid process) was recorded with a three-dimensional motion capture system (NaturalPoint, Inc., Corvallis, OR, United States) at a frequency of 100 Hz (the spatial resolution was less than 0.5 mm). PCs with custom software were used to control the apparatus and record the kinematics.

### Procedure

Each participant was required to place his or her right hand on the starting position before each trial. In this position, the lateral side of the little finger and ulnar palm was touching the surface of the button. Participants were also required to begin each trial with the tips of the thumb and index finger of the right hand touching each other. This pre-trial condition was consistent across every trial of the experiment.

The task in each trial required each participant to reach out to grasp the target object at a comfortable daily life speed and then lift the object about 5 cm. After lifting up the object, the participant put the object back to where it had been and returned his/her hand to the starting position. With respect to grasping (holding) the object, the participants were instructed to approach the object laterally with their fingers and to use the thumb and index finger in such a manner that the line connecting the surface points of each thumb and finger passed through the center of the object’s horizontal circle. Use of the other three fingers was allowed to grasp the target object as long as the thumb and index finger were always the main digits used in the grasping action (Fukui and Inui, 2015).

The vision during the task was manipulated using liquid-crystal shutter goggles. The goggles were opaque before each trial, and participants started their movements after the goggles became transparent. This was accompanied by the experimenter’s voice cue (the “go” signal). Two visual conditions during the movement were tested: an NV condition, in which the goggles closed immediately after the release of the hand from the start switch, and an FV condition, in which the goggles remained transparent during the entire movement (e.g., Fukui and Inui, 2006, 2015). We investigated the effects of the presentation order of these two visual conditions (i.e., visual context) by considering: (i) a blocked condition (separate experimental sessions of trials in FV and NV conditions) and (ii) an alternating condition (sessions of alternating trials of FV and NV conditions) (Whitwell et al., 2008; Tang et al., 2014, 2015).

The experiment comprised two sessions (blocked and alternating), and the order of these sessions was counterbalanced across participants. Each session comprised four sub-sessions, each with 15 trials. In the blocked condition, either the FV or the NV conditions was repeatedly presented during the first two sub-sessions and the other was presented during the last two sub-sessions. Object size was blocked in an ABBA manner. The presentation orders were also counterbalanced across participants; therefore, in addition to the practice trials (fewer than 10 trials), each participant in the groups completed 120 trials [=15 trials × 2 (visual context: blocked, alternating) × 2 (size: 4 cm, 6 cm) × 2 (vision: FV, NV)] across the entire experiment.

### Data Processing and Analysis

The three-dimensional positional data given by Cartesian coordinates from the reflective markers were recorded and filtered offline by a second-order dual-pass Butterworth low-pass filter with a cut-off frequency of 15 Hz. Further offline analysis included computation of wrist velocity, acceleration, and jerk from the filtered position signal. We also calculated grip aperture as the distance between the positions of two reflective markers attached to the thumb and index finger.

Movement onset was defined as the frame in which the tangential velocity first exceeded 50 mm/s, and reach-end time was defined as the frame at which the velocity fell back below this threshold. The typical aperture velocity profile for grasping movements shows positive values throughout the aperture-opening phase until the PGA is achieved; that is followed by negative values as the hand’s fingers close down upon the object. Grasp-end time was defined as the point in time when the negative grip aperture velocity crossed the criteria line (setting -20 mm/s) before returning to approximately 0 mm/s as the fingers made contact with the object.

The time of lifting initiation was defined as the point in time when wrist height velocity exceeded 15 mm/s. Finally, reach duration denoted the time between movement onset and reach-end time, and movement duration was defined as the time between movement onset and grasp-end time (i.e., not including the uplift of the object).

The current study focused on (1) smoothness of reaching movement, (2) grip aperture control according to online vision, and (3) smoothness of chaining motor acts (in this case, uplifting the object after grasping) in autistic participants, compared to typical developing peers. To evaluate these functions, we calculated the following values.

Firstly, the NJS, which is unit-free, was calculated as an index of movement smoothness using the equation shown below (Kitazawa et al., 1993; Teulings et al., 1997). Calculating jerk is acceptable when sampling frequency is around 100 Hz (Yan et al., 2000).

$$\mathrm{NJS}=\sqrt{\frac{1}{2}\int j^{2}(t)dt\times \frac{{\mathrm{RD}}^{5}}{{\mathrm{WD}}^{2}}}$$

In the equation, RD, WD, and *j* denote reach duration, total wrist displacement until reach end, and jerk, respectively. Secondly, the differences of the NJS and PGA between the FV and NV conditions (NJSDiff and PGADiff) were computed to evaluate whether a homogenizing effect induced by visual condition schedule appears in reaching movement smoothness and grip aperture adjustment. Lastly, the difference between the grasp-end time and the time of lifting initiation (DiffGrLf) was calculated as an index of the smoothness of chaining motor acts.

In addition to these above-mentioned values, the values of the transport component (peak wrist velocity, time to peak wrist velocity), the time to PGA, and the reaction time (i.e., the time between the goggles’ opening at the start of the trial and the onset of movement) were measured.

Mean values for each dependent variable (except NJSDiff and PGADiff) were entered into a four-way ANOVA with the group (ASD, TD) as a between-participants factor and the visual context (blocked, alternating), object size (4, 6 cm), and visual condition (FV, NV) as within-participant factors. With respect to NJSDiff and PGAdiff, a three-way ANOVA was applied with the group (ASD, TD) as a between-participants factor and the visual context (blocked, alternating), object size (4, 6 cm) as within-participant factors. If we found an interaction, the simple main effect analysis was examined with the Bonferroni correction.

Furthermore, by pooling the data for the TD and ASD groups, a multiple linear regression and associated stepwise variable selection method were applied to analyze the relationships between the five subcategories of AQ scores (i.e., social skill, attention switching, attention to detail, communication, imagination) and each kinematic parameter to determine whether and which subscale scores would predict the performance of each kinematic parameter.

## Results

**FIGURE 2:**
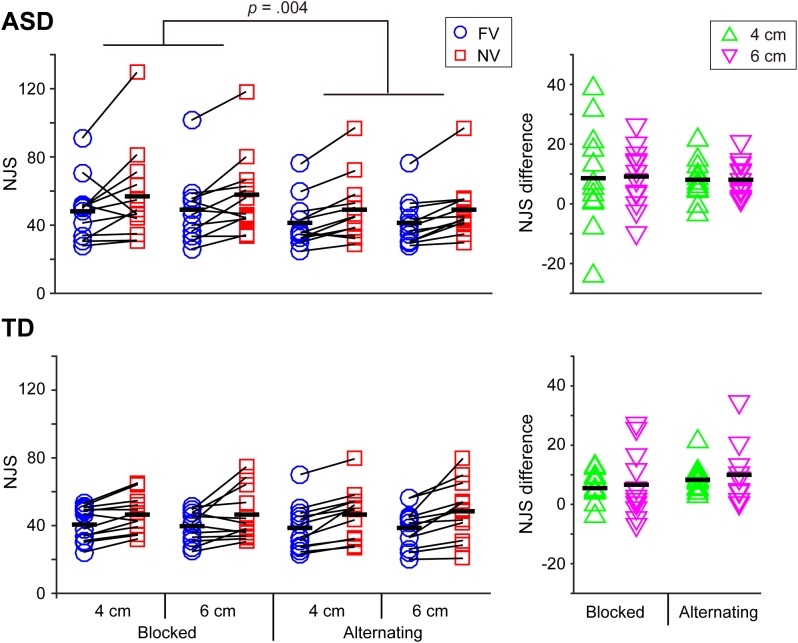
Normalized jerk score (NJS) and the difference in the NJS between the FV and NV conditions in the ASD and TD groups. As for the NJS, a significant interaction between group and visual context was noted and this interaction indicated that the ASD group exhibited a significantly larger value for the blocked condition than for the alternating condition, while the TD group experienced no significant effect of visual context. Black bars indicate mean values in each condition. As for the difference in the NJS, neither significant main effects on factors nor interactions were noted.

**FIGURE 3:**
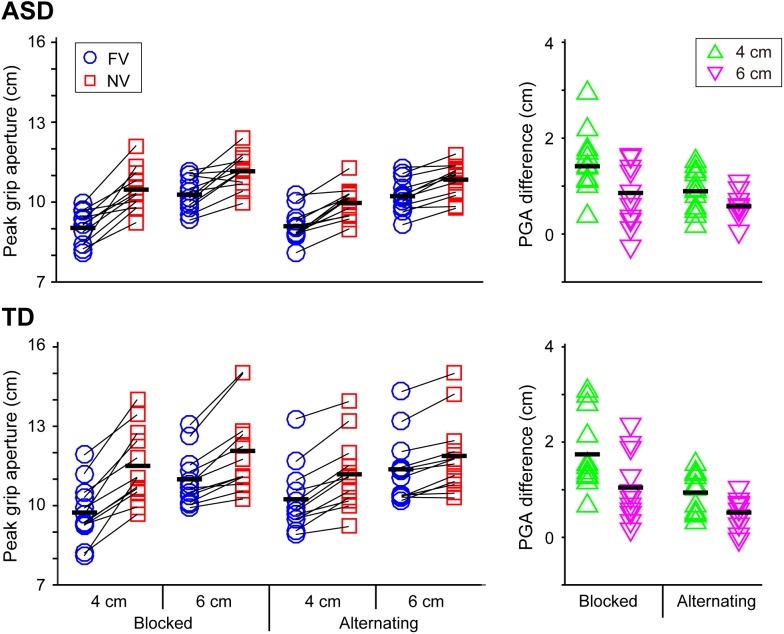
Peak grip aperture (PGA) and the difference in PGA between the FV and NV conditions in the ASD and TD groups. PGA difference between the FV and NV conditions affected by visual context and object size in the ASD group was remarkably similar to those in the TD group. Black bars indicate mean values in each condition.

**FIGURE 4:**
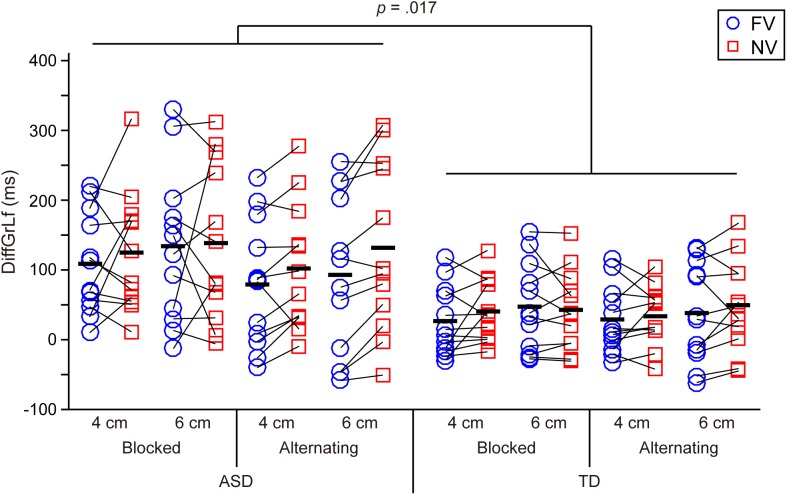
Difference between the grasp-end time and the time of lifting initiation (DiffGrLf) in the ASD and TD groups. Significant main effects of group (i.e., significant longer time in the ASD group than in the TD group) and of object size (i.e., significant longer time for the 6 cm object than for the 4 cm object) were found. Black bars indicate mean values in each condition.

The mean values of kinematic parameters in each experimental condition in the ASD and TD groups were shown in Table 2 and Figures 2–4.

**Table 2:** Mean values (SEs) of the kinematic parameters for each experimental condition in the ASD and TD groups.

	Blocked				Alternating			
		
	4 cm		6 cm		4 cm		6 cm	
				
	FV	NV	FV	NV	FV	NV	FV	NV
**ASD**								
Reaction time (ms)	467	514	501	480	503	525	510	500
	(35)	(71)	(49)	(45)	(68)	(68)	(62)	(54)
Movement duration (ms)	1124	1257	1130	1212	1071	1227	1082	1189
	(50)	(96)	(49)	(84)	(56)	(70)	(54)	(62)
Peak wrist velocity (cm/s)	42.1	40.7	40.9	41.6	45.0	42.9	44.6	42.5
	(2.3)	(3.2)	(2.3)	(3.3)	(2.9)	(3.1)	(2.7)	(3.0)
Time to peak wrist velocity (ms)	446	453	461	445	435	422	445	449
	(22)	(31)	(25)	(31)	(22)	(21)	(23)	(24)
Time to peak grip aperture (ms)	742	760	766	750	712	753	732	763
	(36)	(54)	(41)	(53)	(44)	(43)	(42)	(45)
**TD**								
Reaction time (ms)	403	379	403	386	390	395	399	423
	(34)	(35)	(36)	(32)	(35)	(37)	(35)	(42)
Movement duration (ms)	989	1085	988	1031	963	1104	983	1104
	(54)	(56)	(58)	(58)	(60)	(70)	(59)	(78)
Peak wrist velocity (cm/s)	46.4	47.5	47.7	48	48	46.7	48.7	47.2
	(2.9)	(3.3)	(3.4)	(3.3)	(3.6)	(3.8)	(3.9)	(3.8)
Time to peak wrist velocity (ms)	383	376	385	361	384	390	389	384
	(20)	(24)	(22)	(21)	(25)	(27)	(26)	(24)
Time to peak grip aperture (ms)	677	666	686	667	643	698	667	705
	(46)	(47)	(53)	(45)	(49)	(51)	(51)	(55)


*FV and NV denote full vision and NV conditions during reach-to-grasp movements. 4 and 6 cm are the diameters of the target wooden cylinders. The blocked condition consists of separate experimental sessions of trials under FV and NV conditions and alternating condition consists of sessions of alternating trials of FV and NV conditions.*

### Reaction Time (Table 2)

No significant main effects on factors and no significant interactions were noted (*p* > 0.109).

### Movement Duration (Table 2)

Visual condition was found to have a significant main effect [*F*(1,22) = 28.344, *p* < 0.001, partial η^2^ = 0.563], and the interaction between object size and visual condition was also significant [*F*(1,22) = 8.122, *p* = 0.009, partial η^2^ = 0.270]. The simple main effect analysis revealed that the movement duration in the FV condition was significantly shorter than that in the NV condition for both object sizes.

### Peak Wrist Velocity (Table 2)

No significant main effects on factors and no interactions were noted (*p* > 0.108).

### Time to Peak Wrist Velocity (Table 2)

The interaction between group and object size was significant [*F*(1,22) = 5.196, *p* = 0.033, partial η^2^ = 0.191]. The simple main effect analysis revealed that the time to peak wrist velocity for the 6 cm object was also significantly later than that for the 4 cm object in the ASD group while no significant time difference was found between the 4 and 6 cm objects in the TD group.

### Normalized Jerk Score (NJS) and the Difference in NJS Between the FV and NV Conditions (NJSDiff) (Figure 2)

The main effect of visual condition was significant [*F*(1,22) = 32.846, *p* < 0.001, partial η^2^ = 599], indicating that the NJS in the NV condition was significantly larger than that in the FV condition. The results also showed a significant interaction between group and visual context [*F*(1,22) = 5.021, *p* = 0.036, partial η^2^ = 0.186], as well as a main effect of visual context which indicated that the NJS in the blocked condition was significantly larger than that in the alternating condition [*F*(1,22) = 5.357, *p* = 0.030, partial η^2^ = 0.196]. The simple main effect analysis revealed that the mean NJS in the blocked condition was significantly larger than that in the alternating condition in the ASD group, while there was no significant difference between the blocked and alternating conditions in the TD group.

As far as NJSDiff was concerned, neither significant main effects on factors nor interactions were noted (*p* > 0.345).

### Peak Grip Aperture (PGA) and the Difference in PGA Between the FV and NV Conditions (PGADiff) (Figure 3)

With respect to PGA, significant main effects of group [*F*(1,22) = 6.258, *p* = 0.020, partial η^2^ = 0.222], object size [*F*(1,22) = 279.750, *p* < 0.001, partial η^2^ = 0.927], and visual condition [*F*(1,22) = 120.963, *p* < 0.001, partial η^2^ = 0.846] were found. The results show a larger PGA for the TD group than the ASD group, as well as a larger PGA for the larger object and the NV condition. Significant interactions between visual context and visual condition [*F*(1,22) = 25.094, *p* < 0.001, partial η^2^ = 0.533], and between size and visual condition [*F*(1,22) = 46.464, *p* < 0.001, partial η^2^ = 0.679] were also found. In particular, the simple main effect analysis found that the PGA in the NV condition was significantly larger when performing the task in the blocked condition than when performing it in the alternating condition, while the PGA in the FV condition was significantly larger when performing the task in the alternating condition.

With respect to PGADiff, significant main effects of visual context [*F*(1,22) = 25.094, *p* < 0.001, partial η^2^ = 0.533] and size [*F*(1,22) = 46.464, *p* < 0.001, partial η^2^ = 0.679] were found, indicating that the value in the blocked condition was significantly larger than that in the alternating condition and that the value for the 4 cm object was significantly larger than that for 6 cm object. No significant main effect of group [*F*(1,22) = 0.522, *p* = 0.478] was noted.

### Time to Peak Grip Aperture (Table 2)

A significant interaction between visual context and visual condition was found [*F*(1,22) = 10.036, *p* = 0.005, partial η^2^ = 0.313], indicating a longer time to PGA in the NV condition than in the FV condition when performing the task in the alternating condition. This interaction also denoted that the time to PGA in the alternating condition was significantly earlier than that in the blocked condition when performing the task in the FV condition.

### Difference Between the Grasp-End Time and the Time of Lifting Initiation (DiffGrLf) (Figure 4)

The results showed a significant main effect of group [*F*(1,22) = 6.629, *p* = 0.017, partial η^2^ = 0.232], indicating that the ASD group took significantly longer time than the TD group to begin to lift up the object after the grasp-end time. A significant main effect of size [*F*(1,22) = 5.161, *p* = 0.033, partial η^2^ = 0.190] was also found, indicating that the value for the 6 cm object was significantly longer than that for the 4 cm object.

### Relationship Between Each Kinematic Value and AQ Score

The multiple regression analysis revealed that only the subcategory social skill score was significantly correlated with reaction time (*R*^2^ = 0.172, *p* = 0.044), movement duration (*R*^2^ = 0.416, *p* = 0.043), time to peak velocity (*R*^2^ = 214, *p* = 0.023), and time to PGA (*R*^2^ = 439, *p* = 0.032). We also found that only the subcategory attention switch score was significantly correlated with DiffGrLf (*R*^2^ = 262, *p* = 0.011). The other kinematic values showed no significant correlation with the subcategory AQ scores.

## Discussion

This kinematic study explored whether and how older adolescents and adults with ASD perform reach-to-grasp and uplift movements in comparison to TD peers. Our foci were (1) smoothness of the reaching movement, (2) grip aperture control according to online vision, and (3) smoothness of chaining motor acts.

First, as for the smoothness of the reaching movement, the ASD group showed a significantly larger mean NJS in the blocked schedule than in the alternating schedule, while no significant difference of the mean NJS was shown between these two visual schedule conditions in the TD group. This result indicates that the modulation patterns of reaching movement smoothness according to the visual context itself are different between ASD and TD. Specifically, alternating FV and NV conditions from trial to trial in a session, in contrast to blocking these visual conditions, contributed to reducing the NJS (i.e., increasing reaching movement smoothness) for both FV and NV conditions in the ASD group while visual context had no influence on the NJS in the TD group. At the same time, the main effect of the group (i.e., ASD and TD) on the mean NJS was not significant, suggesting that reaching movement smoothness in the ASD group was comparable to that in the TD group. Furthermore, the homogenizing effect did not operate on the NJS in either the TD or ASD group. This finding strengthens the previous findings by Whitwell et al. (2008), which did not observe the influence of the visual feedback schedule on the transport components they examined.

Second, grip adjustment according to online vision and its context was remarkably similar between ASD and TD peers. Specifically, homogenization in PGA due to visual context (Whitwell et al., 2008; Tang et al., 2014, 2015) occurred in both older adolescents and adults with ASD and their TD peers. This result suggests that sensorimotor memory for grip aperture adjustment was intact (or had been recovered) in the older adolescents and young adults with ASD. Furthermore, in both the TD and ASD groups, the homogenizing effect according to the visual schedule emerged only in grip aperture adjustment (i.e., grasp component), not in reaching movement smoothness (i.e., transport component).

Third, the significantly longer DiffGrLf (i.e., transition period from grasping end to uplift initiation) in the ASD group than in the TD group suggests that ASD participants have difficulties chaining motor acts smoothly and appropriately. It is noteworthy that this significant longer DiffGrLf in ASD group emerged despite no significant difference in reaction time or movement duration between the ASD and TD groups. Namely, the longer transition period from grasping end to uplift initiation in the ASD group cannot be attributed simply to general movement slowness.

As we introduced in the section “Introduction,” Yang et al. (2014) showed that movement duration in school-age children with ASD [mean (SD) age: 7 years 8 months (1 year 4 months)] was longer than those of the TD peers when grasping a small target and that reaching movement in the ASD group is less smooth than that of the TD peers. Furthermore, ASD children cannot use online vision to adjust grip aperture while TD peers appropriately adjust their grip aperture according to the availability of online vision. However, the current study did not find significant differences of movement duration and NJS between the ASD and TD groups. Furthermore, the result of the PGA difference indicated that the ability to adjust grip aperture according to visual schedule in older adolescents and adults with ASD is comparable to those in TD peers. Why the TD group showed a significantly larger PGA than the ASD group must be clarified in a future study. Importantly, the experimental situation of Yang et al. (2014) was much like our current one where the movement speed of the task was comfortable.

With respect to chaining sequential motor acts, Fabbri-Destro et al. (2009) found that children with ASD [mean (SD) age: 10.0 (±2.3)], in contrast to TD children, could not modulate their first action (i.e., reaching to grasp) according to the task difficulty of the second action (i.e., dropping into a container) and argued that ASD children struggle to chain sequential motor acts as an entire action (see also Cattaneo et al., 2007; Forti et al., 2011). The current study simply required participants to perform an uplifting action instead of manipulating the task difficulty after grasping the target object, and Fabbri-Destro et al. (2009) did not calculate the values comparable to our DiffGrLf (i.e., transition period from grasping end to uplift initiation). Therefore, we could not directly compare our current study with Fabbri-Destro et al. (2009), but our results, which showed significantly longer DiffGrLf in the ASD group than in the TD group, and the results of Fabbri-Destro et al. (2009) both demonstrated that ASD participants have difficulties in chaining motor acts.

By comparing the current results concerning older adolescents and adults to previous studies mentioned above concerning school-age children, we can deduce the following developmental trajectories of prehension movements in ASD: First, impairment of reaching smoothness and grip adjustment in school-age children could be compensated by their developmental processes, and their visuo-motor transformation processes would be comparable to TD peers when they grow older (i.e., become older adolescents). By contrast, organizing their sequential motor acts as an entire action is difficult even for older adolescents and adults.

In addition to comparing ASD and TD participants, we investigated the relation between autistic traits and kinematics by pooling the data on the TD and ASD groups. The transition period from grasping end to uplift initiation (DiffGrLf), which showed a significant difference between the ASD and TD groups, was significantly correlated with the subcategory attention switch. Although we found no significant difference between the ASD and TD groups for the following values (except time to peak wrist velocity for the 6 cm condition), the subcategory social skill score showed a (mild) significant correlation with the temporal components (i.e., reaction time, movement duration, time to peak velocity, time to PGA). Although the reason for this specific subcategory’s significant correlations with specific parameters will need to be clarified in future studies, the current results suggest that the AQ subcategory scores could be useful for predicting motor performance for the pooled population of ASD and TD individuals.

The lack of significant differences in parameters between ASD and TD during reach-to-grasp movements (except for time to peak wrist velocity for the 6 cm condition) may be due to the ease of the task in the current experiment, since the target was presented at one fixed location and each target size was blocked in each session. Furthermore, participants were instructed to perform the task at a comfortable daily life speed in the current study (cf. Fukui and Inui, 2006). Of course, the task difficulty would be increased by changing the external and/or internal properties of the target objects (e.g., location, shape, size, etc.) and by increasing the speed of their movement. Such manipulation of task difficulty could result in significant differences in the parameters during reach-to-grasp movements. Furthermore, the relatively small sample size of the current study is a limitation. However, the current findings show that even in simple and comfortable experimental situations older adolescents and adults with ASD exhibited significantly longer transition periods from grasping end to uplift initiation, indicating difficulties in chaining their sequential motor acts as an entire action. This would be the primary cause of autistic individuals’ deficit with regard to understanding others suggested by Fabbri-Destro et al. (2009) and Cattaneo et al. (2007).

Older adolescents and adults with ASD and their TD peers exhibit a similar grip aperture modulation according to online vision and its context in the current experiment, while school-age children cannot perform such a modulation (Yang et al., 2014). That is, like TD peers (Whitwell et al., 2008; Tang et al., 2014, 2015), older adolescents and adults with ASD could appropriately use the predictability of available vision of upcoming trial. Glazebrook et al. (2008) demonstrated that adults with ASD could use advance information, but could not adopt a kind of strategic flexible planning in a manual aiming study. Therefore, what kind of advance (predictive) information could be used (or not used) for individuals with ASD when performing reach-to-grasping movements should be investigated in a future study.

With respect to the effect of visual context on reaching smoothness (NJS), the significant reduction of NJS in the alternating condition compared to the blocked condition was found in the ASD group, while no significant difference in NJS occurred for the TD group. While this latter result for the TD would be due to the ease of the current task (i.e., the floor effect), the increase in reaching smoothness in the alternating condition for the ASD group would support investigations of therapeutic interventions for movement disturbance. For example, it may be better to train motor behaviors across several visual contexts, rather than in fixed visual condition. Although no significant difference in NJS between the ASD and TD groups was found in the current study, future studies using a higher sampling frequency motion capture system in a slightly larger sample size are needed to clarify detailed properties of reaching smoothness.

The current prehension task is simple and performed by one single person. Recently, however, this prehension action was incorporated into a task to investigate the visuomotor processes of joint action carried out concurrently by two people (e.g., Becchio et al., 2008; Sacheli et al., 2013; Curioni et al., 2017). Reasoning about the mind of another person, which is associated with activity in the ventral medial prefrontal cortex (e.g., Castelli et al., 2000; van den Bos et al., 2007), facilitates appropriate joint action. Therefore, both kinematic and neuroimaging studies are required for revealing the mechanism of joint action, and kinematic studies of prehension tasks provide some basis for comparison with kinematic studies of joint action.

In sum, the use of online vision and its context for motor control, which is not fully exploited in school-age children, may be compensated for when individuals with ASD reach late adolescence; however, older adolescents and adults with ASD still have difficulties chaining motor acts.

## Author Contributions

TF conceived, designed, and performed the experiments, analyzed the data, and wrote the manuscript. SK and KN diagnosed the individuals with ASD. MiS confirmed the diagnoses using the Japanese version of the ADOS. AT, MaS, and HA performed psychological the tests. TF, RF, YN, and MW contributed reagents, materials, and analysis tools. All authors discussed the results and commented on the manuscript.

## Conflict of Interest Statement

The authors declare that the research was conducted in the absence of any commercial or financial relationships that could be construed as a potential conflict of interest.
